# Coverage of intermittent preventive treatment of malaria in pregnancy in four sub-Saharan countries: findings from household surveys

**DOI:** 10.1093/ije/dyaa233

**Published:** 2020-12-08

**Authors:** Clara Pons-Duran, Mireia Llach, Charfudin Sacoor, Sergi Sanz, Eusebio Macete, Iwara Arikpo, Máximo Ramírez, Martin Meremikwu, Didier Mbombo Ndombe, Susana Méndez, Manu F Manun’Ebo, Ranto Ramananjato, Victor R Rabeza, Maya Tholandi, Elaine Roman, Franco Pagnoni, Raquel González, Clara Menéndez

**Affiliations:** 1 ISGlobal, Hospital Clínic - Universitat de Barcelona, Barcelona, Spain; 2 CIBER Epidemiología y Salud Pública (CIBERESP), Spain; 3 Centro de Investigação em Saúde de Manhiça (CISM), Maputo, Mozambique; 4 Departament de Fonaments Clínics, Facultat de Medicina, Universitat de Barcelona (UB), Barcelona, Spain; 5 Cross River Health and Demographic Surveillance System, University of Calabar, Cross River State, Nigeria; 6 Bureau d’Étude et de Gestion de l’Information Statistique (BÉGIS), Kinshasa, DRC; 7 Malagasy Associates for Numerical Information and Statistical Analysis (MANISA), Antananarivo, Madagascar; 8 Jhpiego, Affiliate of Johns Hopkins University, Baltimore, MD, USA

**Keywords:** Malaria control, pregnancy, maternal health, sub-Saharan Africa

## Abstract

**Background:**

Intermittent preventive treatment in pregnancy (IPTp) with sulphadoxine-pyrimethamine (SP) is a key malaria prevention strategy in areas with moderate to high transmission. As part of the TIPTOP (Transforming IPT for Optimal Pregnancy) project, baseline information about IPTp coverage was collected in eight districts from four sub-Saharan countries: Democratic Republic of Congo (DRC), Madagascar, Mozambique and Nigeria.

**Methods:**

Cross-sectional household surveys were conducted using a multistage cluster sampling design to estimate the coverage of IPTp and antenatal care attendance. Eligible participants were women of reproductive age who had ended a pregnancy in the 12 months preceding the interview and who had resided in the selected household during at least the past 4 months of pregnancy. Coverage was calculated using percentages and 95% confidence intervals.

**Results:**

A total of 3911 women were interviewed from March to October 2018. Coverage of at least three doses of IPTp (IPTp3+) was 22% and 24% in DRC project districts; 23% and 12% in Madagascar districts; 11% and 16% in Nigeria local government areas; and 63% and 34% in Mozambique districts. In DRC, Madagascar and Nigeria, more than two-thirds of women attending at least four antenatal care visits during pregnancy received less than three doses of IPTp.

**Conclusions:**

The IPTp3+ uptake in the survey districts was far from the universal coverage. However, one of the study districts in Mozambique showed a much higher coverage of IPTp3+ than the other areas, which was also higher than the 2018 average national coverage of 41%. The reasons for the high IPTp3+ coverage in this Mozambican district are unclear and require further study.

**Key Messages**

- Less than a quarter of pregnant women eligible for intermittent preventive treatment of malaria in pregnancy (IPTp) received at least three doses (IPTp3+) in the survey study districts of Madagascar, Nigeria and the Democratic Republic of Congo.
- In these countries’ districts, more than two-thirds of women attending at least four antenatal care visits during pregnancy received less than three doses of IPTp.
- The survey districts of Mozambique had coverage rates of IPTp3+ higher than other study countries and the gap between attendance to antenatal care and uptake of IPTp was lower.

## Background

In sub-Saharan Africa (SSA), it is estimated that 11 million pregnancies were exposed to malaria infection in 2018.^1^^,^^2^ Pregnant women are particularly susceptible to malaria infection, leading to negative consequences for the health of the mother and the offspring, mainly maternal anaemia and low birthweight, and increasing maternal and infant mortality and morbidity.^3–5^

The World Health Organization (WHO) recommends for pregnant women living in malaria endemic areas, the use of long-lasting insecticidal mosquito nets (LLINs) as well as prompt diagnosis and treatment of clinical cases.^1^ Further, in areas with moderate to high malaria transmission in Africa, the WHO has recommended since 1998 the administration of intermittent preventive treatment of malaria in pregnancy (IPTp) with sulphadoxine-pyrimethamine (SP),^6^ the cornerstone of malaria prevention in pregnancy. This consisted in at least two doses of SP after quickening.^6^^,^^7^ However since 2012, IPTp has been recommended at each scheduled antenatal care clinic visit starting in the second trimester of gestation, with the objective of ensuring the uptake of at least three IPTp administrations of SP (IPTp3+).^8^^,^^9^ In 2018, the coverage of IPTP3+ was 31%, which was an improvement compared with previous years (2% in 2010 and 22% in 2017),^2^ but still far from the universal coverage targeted by the WHO.^2^ As of 2020, 36 African countries have adopted the latest recommendation of IPTp.^2^

At the health system level, several factors have been identified as barriers to receiving IPTp or to delivering it by health providers; among these are user fees for antenatal care services and SP delivery, stock-outs of SP, poor knowledge about the IPTp strategy, inability to name SP contraindications and side effects, poor supervision of IPTp uptake at the health facility, high workload of antenatal care staff or lack of cups or drinking water at the health facility.^10–13^

At the user level, several studies have assessed the socioeconomic and demographic factors that may affect IPTp uptake and found that maternal age, gravidity, educational level, occupation, wealth status and residence (rural/urban) were all associated with the uptake of at least two and three doses of IPTp (IPTp2+ and IPTp3+), although how these factors affect IPTp uptake varied across countries.^10^^,^^12^^,^^14–19^

To extend the delivery of IPTp to all women who would benefit from it, the 5-year implementation research project Transforming IPT for Optimal Pregnancy (TIPTOP) was designed with the aim of improving malaria prevention in pregnancy through community distribution of IPTp (C-IPTp) added to standard delivery of IPTp at the antenatal care facilities. Through this project we are implementing and evaluating C-IPTp in four SSA countries with an existing national programme of community health workers and IPTp policy—the Democratic Republic of Congo (DRC), Madagascar, Mozambique and Nigeria.^20^ As part of the project, a cross-sectional study using household surveys was carried out to estimate the baseline coverage of IPTp in the countries’ study areas as part of the evaluation of the impact of C-IPTp after its implementation. Key study indicators collected were the coverage of IPTp3+ and the attendance to antenatal care clinics at least four times during the latest pregnancy. Other study indicators included one or more doses of IPTp (IPTp1+), IPTp2+, one or more antenatal care visits, and attendance to the first antenatal care visit in the first trimester of gestation. In addition, the main factors affecting the uptake of IPTp in the four study countries were assessed.

## Methods

The study protocol was reviewed and approved by the Ethics Review Committee of the World Health Organization (Geneva, Switzerland) [ERC.003009], the Research Ethics Committee of the Hospital Clinic (Barcelona, Spain) [HCB/2017/1062], the Ethics Committee of the Public Health School of the University of Kinshasa (Democratic Republic of Congo) [ESP/CE/047/2017], the Ethical Review Committees of the Ebonyi and Ondo States (Nigeria) [SMOH/47/017 and OSHREC/04/12/2017/032], the Biomedical Research Ethics Committee of the Ministry of Public Health (Madagascar) [122-MSANP-CERBM] and the Institutional Bioethics Committee of the Centro de Investigação em Saúde de Manhiça (Mozambique) [002/2018].

### Study design

The study was a cross-sectional multistage cluster household survey. The sampling, adapted from the Malaria Indicator Surveys and the Expanded Programme on Immunization sampling methods,^21^^,^^22^ was carried out in three stages in each study area, namely: a random selection of clusters using probability proportional to size; a random selection of households using maps and house listing (enumeration of households); and a simple random selection of the women to be interviewed in each household among those meeting the inclusion criteria. Depending on the survey area, 12 to 14 women– were selected to be interviewed per cluster; all clusters in the same survey area had the same sample size. To evaluate C-IPTp after its implementation, cross-sectional surveys will be repeated at mid-term and end-line time points, and their results will be compared with the baseline figures.

### Inclusion criteria

The study population consisted of women of reproductive age—from 13 to 50 years old, depending on country definitions—who had a pregnancy that ended in the 12 months before the interview, who had been resident in the study area for at least 4 months before the end of the pregnancy and who were willing to participate in the survey by signing informed consent/assent, in line with country guidelines. The inclusion of legal minors followed local regulations in each study country.

### Study areas

The study was carried out in two administrative areas in each of the four TIPTOP countries: Kenge and Bulungu Health Zones in DRC, Mananjary and Toliary II districts in Madagascar, Nhamatanda and Meconta districts in Mozambique, and Ohaukwu and Akure South Local Government Areas (LGA) in Nigeria. The eight study areas were heterogeneous in terms of malaria endemicity, urban/rural location and previous estimates of IPTp3+ (Table 1). The IPTp3+ coverage figures shown in Table 1 were used to calculate the sample sizes. These figures were obtained through a review of community-based surveys performed before the start of the project in the study countries.

**Table 1:** Characteristics of study areas

Study areas	Estimates for	Background estimates of IPTp3+ coverage (%)	Malaria prevalence among children 6-59 months old (RDT) (%)	*Plasmodium falciparum* parasite rate in 2-10 year old (%)^a^	Urban/rural location
**DRC**					
Kenge	Kenge Health Zone*/Bandundu former province**/Kwango province***	17.0*	20.2**	16.9***	Rural
Bulungu	Bulungu Health Zone*/Bandundu former province**/ Kwilu province***	12.0*	20.2**	16.1***	Rural
**Madagascar**					
Mananjary	National	10.3	5.1	5.2	Rural
Toliary II	National	10.3	5.1	15.0	Rural
**Mozambique**					
Nhamatanda	Sofala province	36.1	29.4	17.6	Rural
Meconta	Nampula province	19.2	47.9	46.4	Rural
**Nigeria**					
Ohaukwu LGA	Ebonyi state	41.0	51.1	17.9	Rural
Akure South LGA	Ondo state	9.2	21.3	20.9	Urban

Sources: Mozambique: IMASIDA 2015 and IIM 2018; Madagascar: MIS 2016; Nigeria: MIS 2015; DRC :System National d’Information Sanitaire (SNIS) 2016 and EDS 2013–2014.

a Predicted age-standardized parasite rate for *Plasmodium falciparum* malaria for children 2 to 10 years of age for 2017, specific for each study area (except for DRC): The Malaria Atlas Project (Weiss DJ.,2019).

DRC, Democratic Republic of Congo; IPTp3+, three or more doses of intermittent preventive treatment of malaria in pregnancy with sulphadoxine-pyrimethamine; LGA, Local Government Area; RDT, rapid diagnostic tests.

* Estimates for DRC health zones.

** Estimates for DRC former provinces.

*** Estimates for DRC provinces.

### Sample size

To measure with a precision of 5% IPTp3+ coverage rates similar to the previously available estimates (Table 1), it was deemed that 434 and 325 women were needed for Kenge and Bulungu, 284 for Manjanary and Toliary II, 709 and 477 for Nhamatanda and Meconta and 744 and 277 for Ohaukwu and Akure South, respectively—considering a design effect of 2, a 95% confidence interval (CI) and a 10% increase for non-response of the key outcomes.

### Study procedures

Field teams comprised three to five interviewers and one supervisor per study area. All field team members underwent at least five days of intensive training that covered aspects such as, research principles, project objectives and design, study procedures for data collection, detailed descriptions of all questions and use of electronic devices for data collection.

In each cluster, field workers visited all the randomly selected households and checked for the presence of eligible women. If present, household heads were first approached to obtain information about women in reproductive age living in the household. In case the household head was absent, the responsible person of the household at that moment was interviewed. Oral consent to participate in the study was obtained from the household head or responsible person. Once the responsible person gave his/her consent, the study objectives and procedures were explained and the list of eligible women was obtained. Then, the randomly selected woman was requested to sign the informed consent form before proceeding with the interview.

The information collected through the interview included socioeconomic and demographic information of the household and the household head, and the obstetric history and sociodemographic characteristics of the participating woman. The woman’s antenatal care card was always requested, to confirm the information on antenatal care attendance and IPTp uptake. If the antenatal care card was not available, self-reported data were collected. Data were collected using the REDCap Mobile Android App installed on electronic tablets following a direct data entry approach.^23^^,^^24^ The woman’s and household’s questionnaires used to collect the data can be found in Supplementary Material 1, available as Supplementary data at *IJE* online.

### Data management and cleaning

Quality control procedures were put in place during data collection and later with data checking. Errors identified during data collection checking were referred to the interviewers for verification and correction if needed. Data entry was done in real time and rigorous consistency checks were created in order to reduce data entry errors.

If the cluster sample size was exceeded during recruitment in any cluster, only the first 12 to 14 women interviewed were selected for the analysis, according to the time recorded in the tablets for data collection.

### Data analysis

Study outcomes were: the proportion of women having received at least one, two or three doses of IPTp during pregnancy (coverage of IPTp1+, IPTp2+ and IPTp3+); the proportion of women having attended the antenatal care clinic at least once and at least four times; and the proportion of women having attended the first antenatal care clinic visit in the first trimester of gestation. These outcome indicators were derived within the study, using percentages and 95% CI. Due to the differences observed across areas on malaria endemicity, urban/rural location and previous estimates of IPTp3+, as shown in Table 1, all outcomes were analysed by area rather than by country.

A principal multilevel logistic regression model was performed to identify factors associated with IPTp3+ uptake. Secondary multilevel logistic regressions with IPTp2+ and IPTp1+ uptake as outcomes were undertaken to support the results of the principal model. Univariate analyses were carried out to select the independent variables to be included in each of the multivariate models, and only variables showing *P*-values lower than 0.20 were included in the multivariate models (Supplementary Material 2, available as Supplementary data at *IJE* online). Multilevel modelling was selected due to the spatial clustering of the collected data, thus allowing control by the variability across countries and study areas. Cluster levels were country and study area.

The variables that met the criterion to be included in the multivariate regressions were marital status, walking distance from a health facility, schooling, household index, gravidity (only for IPTp3+ as outcome variable), employment status (only for IPTp3+ and IPTp2+ as outcome variables) and sex of the household head (only for IPTp1+ as outcome variable). Analyses were performed using Stata 15 (Stata Corp., College Station, TX, USA).^25^

### Socioeconomic indexes

Two different socioeconomic indexes were generated and were used as potential factors influencing the uptake of IPTp. These indexes were generated using a multiple correspondence analysis (MCA) run for each study area, which is the appropriate methodology to reduce dimensions with categorical data and has been used in similar epidemiological studies.^26^^,^^27^ Four variables related to household’s characteristics were used to generate a household index (materials of the walls and roof, type of toilet and source of water), and 10 to 19 variables pertaining to the possession of assets, depending on the study country, were included in an asset index. The sample of each study area was categorized into three socioeconomic groups (tertiles) based on the continuous distribution of the MCA predicted values.

## Results

From March to October 2018, 3911 women from 297 clusters were interviewed in the study countries (565 in Kenge, 335 in Bulungu, 288 in Mananjary, 284 in Toliary II, 791 in Nhamatanda, 518 in Meconta, 830 in Ohaukwu and 300 in Akure South). Only 12 women per cluster were included in the analysis (except in Ohaukwu where 14 women per cluster were included) following data cleaning procedures explained above. The frequency of participation acceptance was 99.6%.

In all study areas, the arithmetic mean age of participants varied between 25 and 30 years (Table 2). Over 75% of the women had an antenatal care card at the time of the interview, except in Bulungu (DRC) where only 47% of the women had an antenatal care card available. Overall, over 75% of the women reported to have slept under an insecticide-treated net the night before, with the exception of Ohaukwu and Akure South LGAs in Nigeria where this was 37% and 41%, respectively. Giving birth in a health facility ranged from 33% of the women in Mananjary to 93% of the women in Bulungu. Except in Madagascar (Mananjary, 38%, and Toliary II, 51%), and in Mozambique (Meconta, 58%), at least 80% of the deliveries of study participants were attended by skilled health personnel.

**Table 2:** Participant’s characteristics

	DRC		Madagascar		Mozambique		Nigeria	
	Kenge (*n* = 432)	Bulungu (*n* = 323)	Mananjary (*n* = 288)	Toliary II (*n* = 284)	Nhamatanda (*n* = 720)	Meconta (*n* = 480)	Ohaukwu (*n* = 739)	Akure South (*n* = 288)
Reported age in years, mean (SD)	28 (7)	27 (7)	25 (7)	25 (7)	26 (7)	25 (7)	27 (6)	30 (6)
Primigravidae, proportion %	19	21	33	44	24	20	19	27
Time since delivery in days, median (p25–p75)	180 (95–280)	164 (94–282)	172 (100–275)	154 (76–266)	174 (87–272)	166 (70–256)	165 (79–263)	149 (67–262)
Can read, proportion %	59	74	60	70	35	26	76	94
Slept under an ITN the previous night, proportion %	76	76	94	79	91	81	37	41
Gave birth in a health facility, proportion %	80	93	33	48	82	56	68	76
Birth assisted by skilled health personnel, proportion %	80	95	38	51	82	58	80	84
Has antenatal care card, proportion %	76	47	91	82	87	82	88	100

DRC, Democratic Republic of Congo; ITN, insecticide-treated net; SD, standard deviation.

The coverage of IPTp3+ was below 25% in all study areas except in Mozambique where 63% and 35% of the women interviewed in Nhamatanda and Meconta, respectively, had received at least three doses of IPTp in their latest pregnancy (Table 3). Regarding attendance at the antenatal care clinic (Table 4), in all study areas the attendance figures for at least one visit were over 85%. The proportion of women attending the first antenatal care visit during the first trimester of gestation was between 20% and 30% in all areas except in Bulungu (DRC) and Meconta (Mozambique) where it was 6% and 9%, respectively. Attendance for at least four antenatal care visits was generally above 40% and higher than IPTp3+ coverage, except in Meconta where it was 28% in comparison with 35% of IPTp3+ coverage.

**Table 3:** Coverage of IPTp by study area

		IPTp1+	IPTp2+	IPTp3+
		% (95% CI)	% (95% CI)	% (95% CI)
DRC	Kenge (*n* = 432)	66 (62; 71)	44 (39; 44)	22 (18; 26)
	Bulungu (*n* = 323)	64 (59; 70)	47 (42; 53)	24 (19; 29)
Madagascar	Mananjary (*n* = 288)	55 (49; 61)	36 (31; 42)	23 (19; 29)
	Toliary II (*n* = 284)	44 (38; 50)	24 (19; 29)	12 (8; 16)
Mozambique	Nhamatanda (*n* = 720)	95 (93; 96)	81 (77; 83)	63 (60; 67)
	Meconta (*n* = 480)	86 (82; 89)	60 (56; 65)	35 (30; 39)
Nigeria	Ohaukwu (*n* = 739)	33 (30; 36)	22 (19; 25)	11 (9; 14)
	Akure South (*n* = 288)	49 (43; 55)	25 (20; 31)	16 (12; 21)

CI, confidence interval; DRC, Democratic Republic of Congo; IPTp1+/IPTp2+/IPTp3+, one, two and three or more doses of intermittent preventive treatment of malaria in pregnancy.

**Table 4:** Attendance at antenatal care clinic visits by study area

		One or more antenatal care visits	First antenatal care visit before week 13	Four or more antenatal care visits
		% (95% CI)	% (95% CI)	% (95% CI)
DRC	Kenge (*n* = 432)	92 (89; 95)	25 (21; 29)	43 (39; 48)
	Bulungu (*n* = 323)	93 (89; 95)	6 (4; 9)	41 (35; 46)
Madagascar	Mananjary (*n* = 288)	92 (88; 95)	27 (22; 33)	55 (49; 61)
	Toliary II (*n* = 284)	86 (81; 90)	20 (15; 25)	47 (41; 53)
Mozambique	Nhamatanda (*n* = 720)	99 (97; 99)	21 (18; 24)	65 (61; 68)
	Meconta (*n* = 480)	93 (90; 95)	9 (7; 12)	28 (24; 32)
Nigeria	Ohaukwu (*n* = 739)	94 (92; 95)	24 (21; 27)	67 (64; 70)
	Akure South (*n* = 288)	93 (89; 96)	21 (16; 26)	79 (74; 83)

CI, confidence interval; DRC, Democratic Republic of Congo.

**Figure 1:**
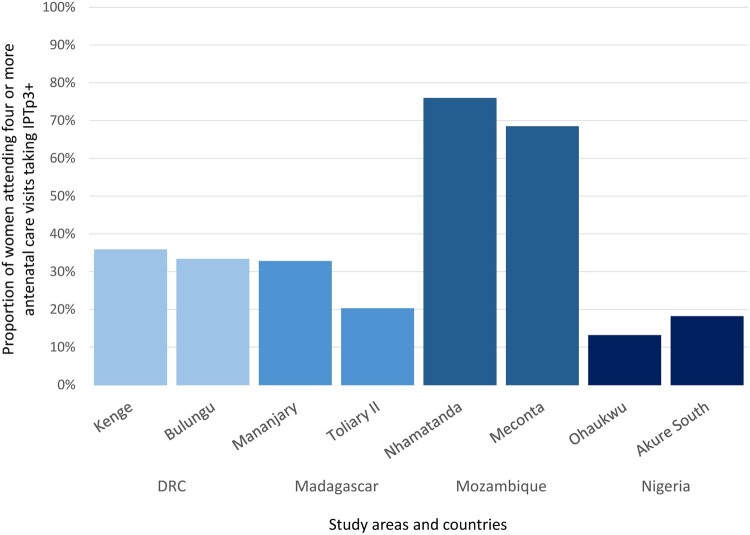
Proportion of women who took three or more doses of IPTp out of those who attended four or more antenatal care visits, per study area. DRC, Democratic Republic of Congo; IPTp, intermittent preventive treatment of malaria in pregnancy

In the Mozambican districts, the proportion of women having received IPTp3+ out of those who attended at least four antenatal care visits was 76% in Nhamatanda and 68% in Meconta (Figure 1). In study areas of Kenge and Bulungu in DRC and in the Mananjary district in Madagascar, only one out of three women who attended at least four antenatal care visits had received at least three doses of IPTp during their last pregnancy. In Toliary II in Madagascar this proportion was 20%, anad in Nigeria it was 13% and 18% in Ohaukwu LGA and Akure South LGA, respectively.

Women with secondary school level of education were more likely to have taken at least three doses of IPTp than women that had never attended school [odds ratio (OR) 1.77, 95% CI 1.34; 2.33, *P*-value <0.0001) (Table 5). Multigravidae were less likely to have taken at least three doses of IPTp during their latest pregnancy than primigravidae (OR 0.80, 95% CI 0.65; 0.98, *P*-value = 0.03). The other factors included in the multivariate analysis with IPTp3+ as main outcome showed wide 95% CIs, leading to imprecise estimates, and including the possibility of no association between these factors and IPTp3+.

**Table 5:** Multilevel logistic regression models with IPTp3+ as outcome variable

**Variable** ^a^		Multilevel univariate models		Multilevel multivariate model	
		OR (95% CI)	*P*-value	OR (95% CI)	*P*-value
Reported age (years) (*n* = 3474)		0.99 (0.98; 1.01)	0.33	–	–
Marital status (*n *= 3476)	Married or in union	1	0.15	1	0.43
	Single (never married)	0.77 (0.58; 1.02)		0.80 (0.57; 1.12)	
	Separated, divorced, widowed	1.13 (0.76; 1.69)		0.92 (0.53; 1.61)	
Sex of the household head (*n* = 3468)	Female	1	0.27	–	–
	Male	1.14 (0.90; 1.43)		–	
Walking distance to the health facility (*n* = 3366)	<60 min	1	0.15	1	0.36
	≥60 min	0.88 (0.74; 1.05)		0.92 (0.76; 1.10)	
Gravidity (*n* = 3453)	Primigravidae	1	0.01	1	0.03
	Multigravidae	0.79 (0.65; 0.95)		0.80 (0.65; 0.98)	
Schooling (*n* = 3215)	None	1	<0.0001	1	< 0.0001
	Primary	1.28 (1.02; 1.60)		1.22 (0.97; 1.55)	
	Secondary or higher	1.95 (1.50; 2.54)		1.77 (1.34; 2.33)	
Employment status (*n *= 3479)	Not working nor studying	1	0.06	1	0.23
	Working or studying	0.8 (0.64; 1.01)		0.86 (0.67; 1.10)	
Whether the woman is the household head (*n *= 2917)	No	1	0.50	–	–
	Yes	1.10 (0.83; 1.47)		–	
Household index (*n *= 3479)	Poorest	1	0.04	1	0.16
	Intermediate	0.85 (0.69; 1.03)		0.90 (0.73; 1.10)	
	Wealthiest	1.09 (0.89; 1.33)		1.10 (0.89; 1.36)	
Assets index (*n *= 3479)	Poorest	1	0.43	–	–
	Intermediate	0.96 (0.79; 1.17)		–	
	Wealthiest	0.88 (0.72; 1.07)		–	

CI, confidence interval; IPTp3+, three or more intermittent preventive treatment doses; OR, odds ratio.

a The first listed category of each variable has been taken as reference value.

Women with secondary education level were more likely to have taken at least one or two doses of IPTp than those without any schooling (OR 1.53, 95% CI 1.16; 2.02, *P*-value <0.01; OR 1.59, 95% CI 1.24; 2.04, *P*-value <0.001, respectively, for IPTp1+ and IPTp2+) (Supplementary Materials 3 and 4, available as Supplementary data at *IJE* online). The household socioeconomic index was also associated with both IPTp1+ and IPTp2+ uptake, showing that women in the intermediate socioeconomic group were less likely to have taken one or more doses of IPTp (OR 0.68, 95% CI 0.55; 0.83, *P*-value <0.001) or two or more doses of IPTp (OR 0.74, 95% CI 0.61; 0.89, *P*-value <0.01) during their latest pregnancy than women in the first socioeconomic group (the poorest in their respective study areas). No differences were found between women in the highest and lowest socioeconomic tertiles. The other factors included in the multivariate analyses showed wide 95% CIs, leading to imprecise estimates and including the possibility of no association between these factors and IPTp (Supplementary Materials 3 and 4).

## Discussion

This study reports the baseline estimates of IPTp3+ coverage in four SSA countries where community delivery of IPTp is being evaluated as part of the TIPTOP project. This assessment was conducted before the implementation of a community IPTp programme and followed a robust methodology for estimating coverage at district level, which complements available national estimates.

The uptake of IPTp3+ was found to be less than 25% in three out of the four study countries, namely DRC, Madagascar and Nigeria. Unexpectedly, in Mozambique the estimates of IPTp3+ uptake were considerably higher, especially in Nhamatanda district where it was 63%.

In Mozambique, recent estimates from the Demographic and Health Surveys (DHS) showed an increasing trend on IPTp3+ uptake.^28^ In 2018, in Sofala province—where Nhamatanda district lies—the DHS IPTp3+ indicator was 48%,^28^ lower than the figure we observed in Nhamatanda district (63%). The same year in northern Nampula province—where Meconta is located—the DHS estimate for IPTp3+ was 39%, similar to the 35% we found in Meconta district in the study household surveys. Potential explanations for the higher coverage rate of IPTp3+ in Nhamatanda district include the presence of several stakeholders and non-governmental organizations focused on maternal health and malaria prevention.^29^ In Nigeria, most recent estimates (2018) from Ebonyi State—where Ohaukwu LGA is located—showed a decrease in IPTp3+ uptake from 2015, whereas Ondo State—that includes Akure South LGA—experienced an increase in recent years.^30^ Figures at the state level are much higher than the present household survey estimates for LGA in both cases.^30^ For Madagascar and DRC, 2016 estimates at national (Madagascar) and health zone level (DRC) were lower than the coverages found in this study.^31^^,^^32^ These findings suggest that accurate evaluation of this intervention for policy guidance requires IPTp coverage to be estimated at the lowest possible territorial level and, whenever possible, to be site specific, since national (and even provincial) level estimates may not always reflect actual coverage in subnational areas.

Regarding attendance to at least four antenatal care visits, estimates were greater than 40% in all areas. The low proportion of women who took IPTp3+ over those who attended four or more antenatal care visits indicates that some women attend antenatal clinics without receiving IPTp; this was especially the case in Madagascar, DRC and Nigeria (Figure 1). In the four countries, part of the difference between antenatal care attendance and IPTp uptake might be explained by a higher proportion of women attending the antenatal clinic early, since IPTp is not administered during the first trimester of pregnancy.^33^ Nevertheless, our findings show that the proportion of women attending the first antenatal care visit in the first trimester is generally low (less than a quarter of the survey’s sample). The finding of a mismatch between antenatal care attendance and IPTp uptake has been reported earlier.^10^^,^^12^^,^^18^^,^^34–36^

The reasons for the suboptimal indicators' coverages and the mismatch between antenatal care attendance and IPTp administrations could be explained by contextual factors that we did not measure in this survey. Some factors that have been found to negatively affect both antenatal care attendance and IPTp uptake may be: poor infrastructures at the health facility (e.g. lack of water at the health facilities or lack of clean and comfortable examination rooms); difficult access to health facilities (due to difficult climate conditions, lack of transportation infrastructures or political instability); SP stock-outs; and negative attitudes of the health personnel towards providing IPTp.^37^^,^^38^

Women’s education was found to increase IPTp uptake. The observed association between IPTp and school level was robust and consistent across the three analytical models. These findings are in line with previous reports and a meta-analysis, which reported associations between education level and an increase in IPTp2+ uptake.^10^^,^^12^^,^^14^^,^^18^^,^^19^ This observation supports the use of a community-based strategy of IPTp-SP delivery, since the current distribution of the IPTp3+ indicator is inequitable among women with different educational levels.

The finding of this study regarding maternal gravidity is in line with that of a meta-analysis indicating that primigravid women are more likely to take IPTp than multigravid women.^10^ However, this finding was not consistent across the three models built; only the increase in IPTp3+ uptake was associated with first pregnancy. Other variables such as socioeconomic status, knowledge of malaria and use of additional malaria preventive measures were also found to affect the uptake of IPTp2+ in previous studies.^10^ In this study, socioeconomic status was found to be associated with increased uptake of IPTp2+ and IPTp1+, although the results are difficult to interpret since the average population in terms of wealth was more likely to take IPTp than the poorest and the wealthiest groups. This could be explained by a limited variability in the socioeconomic status of the sampled women which was not captured by the household and asset indexes. We did not find IPTp3+ uptake to be associated with the socioeconomic status of the women.

This study has two main limitations. First, the potential recall bias in some variables, such as antenatal care attendance and IPTp uptake, which we think is reduced by extracting the information from the antenatal care card in the majority of cases. With the exception of women in DRC where a low proportion had an antenatal care card at the time of the interview (76% in Kenge, 46% in Bulungu), the majority of women interviewed had an antenatal care card (more than 80% in the other study countries). Second, the same questions were included in the household surveys to generate the socioeconomic indexes in the eight study districts; this implies that the indexes created may not have captured adequately the differences among women of diverse socioeconomic backgrounds at each study site.

On the other hand, this study has several strengths that conferred relevance to the results. This was a multicountry study, with a large sample size per study district making the observed estimates robust and district specific. In addition, the pooled multicountry regression findings have a high external validity since they were obtained by analysing data from eight different districts from four countries, and they were consistent across the three models built. This facilitates the extrapolation of the regression results to other SSA countries.

In conclusion, despite continued call to actions in favour of malaria prevention in pregnancy, findings on IPTp3+ uptake in Madagascar, Nigeria, DRC and Mozambique were lower than the global target of ensuring universal coverage for malaria prevention.^2^ However, although still far from that, Nhamatanda district in Mozambique out-performed the other project areas. These results underscore the need to explore complementary strategies, such as C-IPTp, to improve malaria control in pregnancy

## Supplementary Data

Supplementary data are available at *IJE* online.

## Funding

This work was supported by: UNITAID [2017–13-TIPTOP] through a sub-award signed with Jhpiego [17-SBA-101]; the Spanish Ministry of Science and Innovation through the ‘Centro de Excelencia Severo Ochoa 2019–2023’ Program [CEX2018-000806-S]; the Generalitat de Catalunya through the CERCA Program; and the Spanish Ministry of Education and Vocational Training [FPU15/03548 to C.P.D.].

## Supplementary Material

dyaa243_Supplementary_Data
