# Supplementary material for: Coverage of intermittent preventive treatment of malaria in pregnancy in four sub-Saharan countries: findings from household surveys
Source: Int J Epidemiol. 2020 Dec 8;50(2):550–9. doi: 10.1093/ije/dyaa233 (PMC8128463; doi:10.1093/ije/dyaa233)
Supplement: dyaa243_Supplementary_Data [file dyaa243_supplementary_data.zip › ije-2020-03-0472-File008.pdf]

Supplementary material 1. Household and woman's questionnaires

Household Survey part 1
Household questionnaire

	SECTION 1: GENERAL INFORMATION	
1.	Country	DRC ☐ Madagascar ☐ Mozambique ☐ Nigeria ☐
2.	District/Area ID	|__|__|
3.	Cluster ID	|__|__|
4.	Household ID	|__|__|__|
5.	GPS coordinates	[The mobile device records this data]
6.	Is the household head available?	Yes ☐ No/Not found ☐ Empty Household ☐<br>*If "Empty Household", go to the next selected Household*
7.	Does the household head accept to proceed with the interview?	Yes ☐ No ☐<br>*If "No", answer Q8 and Q9 and go to the next household*
8.	Household head/interviewee initials	|__|__|__|__|
9.	Sex of the household head	Female ☐ Male ☐
10.	Are there women of childbearing age [*country specific definition]* currently living in the household?<br>*(Note: Household=A group of people who live and eat together, sharing the same cooking pot/space/kitchen*)<br>*If "No", go to the next Household*<br>*If "Yes", complete the Household Control Sheet with the help of the Household head and answer questions 10.1, 10.1.1 and 10.1.1.1*	Yes ☐ No ☐
	10.1 How many women of childbearing age are currently living in the household?<br>*If the answer is "0", close the survey and proceed with the following Household of your list.*	|__|__|
	10.1.1 How many women of childbearing age [*country specific definition]* have had a pregnancy that ended during the last 12 months before the interview? (*Inclusion Criteria 1*)	|__|__|
	10.1.1.1 How many of them* have been resident in the Household for at least 4 months before the end of the pregnancy? (*Inclusion Criteria 2*)<br>(*Them: women of childbearing age that have had a pregnancy that ended during the last 12 months before the interview)<br>*If question 10.1.1.1 is higher or equal than 1, select one eligible woman from the Household control sheet, otherwise go to the next household*<br>*[NOTE: Eligible woman= woman living in the household that meets both criteria 1 AND criteria 2]*	|__|__|
	10.1.2 Has de selected woman agreed/assent to sign de informed consent?	Yes ☐ No ☐
	10.1.2.1 *If "No"*, why?	She has refused ☐<br>She is unable to respond ☐<br>She is not available ☐<br>Other ☐<br>Specify other: ____________________________

SECTION2: SOCIO-ECONOMIC CHARACTERISTICS OF HOUSEHOLD HEAD

11.	What is the HIGHEST level of school *you/the Household Head* have attended? [*country specific categories*]	None ☐<br>Primary ☐<br>Secondary ☐<br>Higher ☐
12.	What is *your/the Household Head* MAIN occupation outside the household? [*country specific categories*]	Domestic ☐<br>Farmer ☐<br>Student ☐<br>Business ☐<br>Services ☐<br>None ☐<br>Other ☐<br>Specify other________________
13.	What is *your/the Household Head* MAIN type of income? [*country specific categories*]	Paid employment (Full time) ☐<br>Paid employment (seasonal) ☐<br>Self-employment ☐<br>In-kind ☐<br>No salary ☐

SECTION 3: CHARACTERISTICS OF THE HOUSEHOLD

14.	What is the roof of the main construction made with? [*country specific categories*] *(Multi-choice)*	Metal sheet ☐<br>Grass ☐<br>Reeds ☐<br>Other ☐<br>Specify other ________________
15.	What are the walls of the main construction made with? [*country specific categories*] *(Multi-choice)*	Mud plastered ☐<br>Bricks and adobe ☐<br>Bricks and cement ☐<br>Reeds ☐<br>Metal sheets ☐<br>Other ☐<br>Specify other ________________
16.	What is the main source of drinking water used by the household? [*country specific categories*]	Borehole/hand pump ☐<br>Protected Shallow well ☐<br>Open shallow well ☐<br>Protected spring ☐<br>River /Stream ☐<br>HH connection / Stand pipe /Tanker ☐<br>Dam / Pon ☐<br>Other ☐<br>Specify other ________________
17.	What type of toilet do you have in the household? [*country specific categories*]	Flush / pour flush ☐<br>Pit latrine without a slab ☐<br>Pit latrine with a slab ☐<br>Hanging toilet ☐<br>Does not have toilet/latrine in the household ☐<br>Other ☐<br>Specify other ________________
18.	The household has? (multiple choice) [*country specific categories*]	Electricity ☐<br>Cell phone ☐<br>Television ☐<br>Bicycle ☐<br>Computer ☐<br>Fridge ☐<br>Car / Motorbike ☐<br>Stove ☐

Household Survey part 2

Woman's questionnaire

SECTION 1: INCLUSION CRITERIA CHECK

1. When has your last pregnancy ended? |__|__|/|__|__|/|__|__|__|__| (Date: dd/mm/yyyy)

2. Has the pregnancy ended within the 12 months before the date of the interview? *(Inclusion Criteria 1)* Yes ☐ No ☐

3. Have you been resident in [*name of TIPTOP project intervention area*] for at least 4 months before the end of the pregnancy? *(Inclusion Criteria 2)* Yes ☐ No ☐

4. Has she signed the informed consent? *(Inclusion Criteria 3)* Yes ☐ No ☐

If the answer is "No", answer Q4.1, close the questionnaire and proceed to the next Household

4.1 *If "No"*, why?
- She has refused ☐
- She is unable to respond ☐
- She is not available ☐
- Other ☐

Specify other: ___________________________

IF SHE MEETS THE 3 CRITERIA, PROCEED WITH THE INTERVIEW OTHERWISE GO TO THE NEXT HOUSEHOLD

SECTION 2: MATERNAL HEALTH SECTION

5. How old are you? (years) |__|__|

If no ID card, use a historical calendar

6. Have you attended the ANC clinic during your last pregnancy? Yes ☐ No ☐

6.1 If the answer is "No", why? *(multi choice)*
- I was not aware of ANC service ☐
- The health facility is too far ☐
- The transport to the health facility is too expensive ☐
- I think ANC services are not important ☐
- ANC services are not good quality ☐
- I do not trust ANC services ☐
- My husband advised me not to go ☐
- The family advised me not to go ☐
- I consulted a traditional practitioner instead ☐
- The Community advised me not to go ☐
- Other reason ☐

Specify other_________________________________

If the answer is yes:

6.1 Do you have the ANC card? Yes ☐ No ☐

If the answer is YES, 6.1.1 take a photo of the ANC card

If the answer is NO,

6.1.1 why not?
- ANC card is lost/damaged ☐
- I was not given one ☐
- Someone else keeps it ☐

If so, who? ________________
- Other ☐

Specify other_________________________________

	6.2 How many weeks pregnant were you when you first attended the ANC clinic? *(check ANC card if she has it)*	|__|__|
	6.3 Was the first attendance before quickening?	Yes ☐ No ☐
	6.4 How many times did you attend ANC clinic during your last pregnancy? (*cross-check with ANC card if available)*	|__|__|
7.	How many times have you been seen by a health provider [*country specific definitions]* during your last pregnancy including ANC?	|__|__|
8.	Do you know about the SP service provided in your community by community health workers [*community specific name*] to keep you from getting malaria?	Yes ☐ No ☐
9.	Have you taken SP [*Country specific commercial name*] during last pregnancy to keep you from getting malaria?<br><br>*If the answer is yes,*	Yes ☐ No ☐ Don't know ☐
	9.1 How many times have you taken it?<br>*(Reminder: we are considering full treatment, not number of tablets)*	|__|__|
	9.1.1 Have you got it at the Health Facility? Yes ☐ No ☐ Don't know ☐ If yes, how many times? |__| 9.1.2 Have you got it from the community health workers? Yes ☐ No ☐ Don't know ☐ If yes, how many times? |__| 9.1.3 Have you got it at a Private pharmacy? Yes ☐ No ☐ Don't know ☐ If yes, how many times? |__| 9.1.4 Have you got it in another place? Yes ☐ No ☐ Don't know ☐ If yes, how many times? |__| <br>Specify other ________________________________	
10.	Have you been ill with fever at any time during your last pregnancy? Yes ☐ No ☐ Don't know ☐<br>*If YES,*<br>10.1 How many times? |__| 10.2 Have you gone to the clinic? Yes ☐ No ☐<br>10.2.1 If yes, which medicine have they given to you to treat this fever? *(multiple choice)*<br>**Antimalarial drugs**<br>Artemisinin Combination therapy (ACT) ☐<br>Amodiaquine ☐<br>SP/Fansidar ☐<br>Artesunate iv ☐<br>Artmeter Injection ☐<br>Quinine ☐<br>Other antimalarial drug ☐<br>*Specify other*____________<br>**Non-antimalarial drugs** ☐<br>*Specify* _________________<br>**I wasn't given any treatment** ☐	
11.	Are you taking Cotrimoxazole (CTX)? *(show Picture)*	Yes ☐ No ☐ Don't know ☐
12.	Are you taking antiretroviral therapy (ART)? *(show Picture)*	Yes ☐ No ☐ Don't know ☐
13.	Have you received an ITN during your last pregnancy?<br>*If the answer is YES,*<br>13.1 Did you obtain the ITN in the ANC clinic?	Yes ☐ No ☐<br><br>Yes ☐ No ☐
14.	Have you slept under an ITN last night?	Yes ☐ No ☐
15.	How many children were you bearing in your last pregnancy?	|__|__|

16.	What has been the outcome of your last pregnancy?	Number of Live births |__| Number of Stillbirths |__| Number of Spontaneous abortions |__|
	If any live birth or stillbirth proceed with Q16.1, otherwise go to Q17	
	16.1 Where have you given birth?	Health facility ☐<br>Home ☐<br>On the way to the Heath facility ☐<br>Other ☐<br>Specify other ________________
	16.2 Who has assisted you with the delivery? [*country specific definitions*]	Skilled ☐<br>Non-skilled ☐<br>Nobody, I delivered alone ☐
17.	How many months pregnant were you when your last pregnancy ended?	|__|

	**SECTION 3: OBSTETRIC HISTORY**	
18.	How many times have you been pregnant including the pregnancy that has ended within the last 12 months?	|__|__|
19.	Are you currently pregnant?	Yes ☐ No ☐

	**SECTION 4: SOCIO-ECONOMIC AND DEMOGRAPHIC CHARACTERISTICS OF THE PARTICIPANT**	
20.	Are you the household head?	Yes ☐ No ☐
	20.1 What is the HIGHEST level of school that you have attended? [*country specific categories]*	None ☐<br>Primary ☐<br>Secondary ☐<br>Higher ☐
	20.2 What is your MAIN occupation outside the household? [*country specific categories]*	Domestic ☐<br>Farmer ☐<br>Student ☐<br>Business ☐<br>Services ☐<br>None ☐<br>Other ☐<br>Specify other ________________
	20.3 What is your MAIN type of income? [*country specific categories]*	Paid employment (Full time) ☐<br>Paid employment (seasonal) ☐<br>Self-employment ☐<br>In-kind ☐<br>No salary ☐
21.	Can you read? *(ask her to read a sentence)*	Yes ☐ No ☐
22.	Can you write?	Yes ☐ No ☐
23.	At what health facility do you usually go to seek treatment?	[*District specific list of health facilities*]
24.	By which means do you usually go to the health facility?	Walking ☐<br>Private Car ☐<br>Public transport ☐<br>Other ☐<br>Specify other ________________
25.	How long does it take you to reach the nearest health facility by foot?	|__|__|hours |__|__|minutes
26.	What is your religion?	*[Country specific categories]*

27. What is your ethnic group? [*Country specific categories*]

28. What is your marital status?

- Single (never married) ☐
- Married or in union ☐
- Separated, but still legally married ☐
- Divorced ☐
- Widow ☐

If married or in union,

28.1 Is your husband/partner the head of the household? Yes ☐ No ☐

28.1.1 What is the HIGHEST level of school attended by your husband/partner? [*country specific categories*]

- None ☐
- Primary ☐
- Secondary ☐
- Higher ☐

28.1.2 What is the MAIN occupation of your husband/partner? [*country specific categories*]

- Domestic ☐
- Farmer ☐
- Student ☐
- Business ☐
- Services ☐
- None ☐
- Other ☐

Specify other ________________________________

28.1.3What is the MAIN Type of income of your husband/partner? [*country specific categories*]

- Paid employment (Full time) ☐
- Paid employment (seasonal) ☐
- Self-employment ☐
- In-kind ☐
- No salary ☐

INTERVIEWER INFORMATION

29. Interviewer ID |__|__|__|__|__|

30. Date of the interview |__|__|/|__|__|/|__|__|__|__| (dd/mm/yyyy)
