# Supplementary material for: Coverage of intermittent preventive treatment of malaria in pregnancy in four sub-Saharan countries: findings from household surveys
Source: Int J Epidemiol. 2020 Dec 8;50(2):550–9. doi: 10.1093/ije/dyaa233 (PMC8128463; doi:10.1093/ije/dyaa233)
Supplement: dyaa243_Supplementary_Data [file dyaa243_supplementary_data.zip › ije-2020-03-0472-File009.docx]

**Supplementary material 2. Categorization of potential factors affecting uptake of IPTp.**

| **Factors/Variables** | **Categories** |
| --- | --- |
| **Age of the woman at the moment of the interview** | Continuous variable |
| **Schooling** | No education  Primary education  Secondary education  Higher education |
| **Occupation** | None  Working/studying |
| **Household index** | 1^st^ group (the poorest)  2^nd^ group  3^rd^ group (the wealthiest) |
| **Asset index** | 1^st^ group (the poorest)  2^nd^ group  3^rd^ group (the wealthiest) |
| **Marital status** | Single/never in union  Married/in union  Separated/divorced/widowed |
| **Whether the woman is the household head** | Yes  No |
| **Sex of the household head** | Female  Male |
| **Walking distance to the nearest health facility** | ≤60 min  > 60 min |
| **Gravidity** | Primigravidae  Multigravidae |

***Notes:*** *IPTp – intermittent preventive treatment of malaria in pregnancy.*
