# Supplementary material for: Coverage of intermittent preventive treatment of malaria in pregnancy in four sub-Saharan countries: findings from household surveys
Source: Int J Epidemiol. 2020 Dec 8;50(2):550–9. doi: 10.1093/ije/dyaa233 (PMC8128463; doi:10.1093/ije/dyaa233)
Supplement: dyaa243_Supplementary_Data [file dyaa243_supplementary_data.zip › ije-2020-03-0472-File010.docx]

**Supplementary material 3. Multi-level logistic regression models with IPTp2+ as outcome variable**

| **Variable** | | **Multi-level univariate models** | | **Multi-level multivariate model** | |
| --- | --- | --- | --- | --- | --- |
|  |  | **OR (95% CI)** | **p-value** | **OR (95% CI)** | **p-value** |
| **Reported age (years)** [n=3474] | | 1 (0.99 – 1.01) | 0.97 | – | – |
| **Marital status** [n=3476] | Married or in union | 1 | 0.06 | 1 | 0.38 |
|  | Single (never married) | 0.76 (0.58 – 0.98) |  | 0.82 (0.60 – 1.12) |  |
|  | Separated, divorced, widowed | 1.23 (0.82 – 1.83) |  | 1.18 (0.68 – 2.04) |  |
| **Sex of the household head** [n=3468] | Female | 1 | 0.26 | – | – |
|  | Male | 1.13 (0.91 – 1.40) |  | – |  |
| **Walking distance to the health facility** [n=3366] | <60 min | 1 | 0.06 | 1 | 0.26 |
|  | ≥60 min | 0.85 (0.73 – 1.01) |  | 0.9 (0.76 – 1.08) |  |
| **Gravidity** [n=3453] | Primigravidae | 1 | 0.71 | – | – |
|  | Multigravidae | 0.97 (0.81 – 1.15) |  | – |  |
| **Schooling** [n=3215] | None | 1 | <0.0001 | 1 | <0.001 |
|  | Primary | 1.26 (1.01 – 1.57) |  | 1.22 (0.97 – 1.53) |  |
|  | Secondary or higher | 1.72 (1.35 – 2.20) |  | 1.59 (1.24 – 2.04) |  |
| **Employment status** [n=3479] | Not working nor studying | 1 | 0.02 | 1 | 0.06 |
|  | Working or studying | 0.74 (0.58 – 0.95) |  | 0.78 (0.60 – 1.01) |  |
| **Whether the woman is the household head** [n=2917] | No | 1 | 0.52 | – | – |
|  | Yes | 1.09 (0.83 – 1.44) |  | – |  |
| **Household index** [n=3479] | Poorest | 1 | <0.01 | 1 | <0.01 |
|  | Intermediate | 0.76 (0.63 – 0.91) |  | 0.74 (0.61 – 0.89) |  |
|  | Wealthiest | 0.96 (0.80 – 1.16) |  | 0.96 (0.79 – 1.18) |  |
| **Assets index** [n=3479] | Poorest | 1 | 0.37 | – | – |
|  | Intermediate | 0.91 (0.76 – 1.09) |  | – |  |
|  | Wealthiest | 0.88 (0.74 – 1.06) |  | – |  |

**The first listed category of each variable has been taken as reference value.*

***Note****: CI – confidence interval; IPTp2+ – two or more intermittent preventive treatment doses; OR – odds ratio.*
