# Supplementary material for: Coverage of intermittent preventive treatment of malaria in pregnancy in four sub-Saharan countries: findings from household surveys
Source: Int J Epidemiol. 2020 Dec 8;50(2):550–9. doi: 10.1093/ije/dyaa233 (PMC8128463; doi:10.1093/ije/dyaa233)
Supplement: dyaa243_Supplementary_Data [file dyaa243_supplementary_data.zip › ije-2020-03-0472-File011.docx]

**Supplementary material 4. Multi-level logistic regression models with IPTp1+ as outcome variable**

| **Variable** | | **Multi-level univariate models** | | **Multi-level multivariate model** | |
| --- | --- | --- | --- | --- | --- |
|  |  | **OR (95% CI)** | **p-value** | **OR (95% CI)** | **p-value** |
| **Reported age (years)** [n=3474] | | 1 (0.99 – 1.01) | 0.89 | – | – |
| **Marital status** [n=3476] | Married or in union | 1 | 0.09 | 1 | 0.08 |
|  | Single (never married) | 0.75 (0.57 – 0.99) |  | 0.67 (0.47 – 0.95) |  |
|  | Separated, divorced, widowed | 0.79 (0.50 – 1.24) |  | 0.90 (0.47 – 1.73) |  |
| **Sex of the household head** [n=3468] | Female | 1 | 0.05 | 1 | >0.9999 |
|  | Male | 1.26 (1.00 – 1.58) |  | 1.00 (0.70 – 1.42) |  |
| **Walking distance to the health facility** [n=3366] | <60 min | 1 | 0.08 | 1 | 0.11 |
|  | ≥60 min | 0.85 (0.72 – 1.02) |  | 1.86 (0.71 – 1.04) |  |
| **Gravidity** [n=3453] | Primigravidae | 1 | 0.31 | – | – |
|  | Multigravidae | 1.10 (0.91 – 1.32) |  | – |  |
| **Schooling** [n=3215] | None | 1 | <0.0001 | 1 | <0.01 |
|  | Primary | 1.24 (0.96 – 1.60) |  | 1.13 (0.86 – 1.47) |  |
|  | Secondary or higher | 1.75 (1.34 – 2.29) |  | 1.53 (1.16 – 2.02) |  |
| **Employment status** [n=3479] | Not working nor studying | 1 | 0.31 | – | – |
|  | Working or studying | 0.84 (0.61 – 1.17) |  | – |  |
| **Whether the woman is the household head** [n=2917] | No | 1 | 0.95 | – | – |
|  | Yes | 1.01 (0.74 – 1.38) |  | – |  |
| **Household index** [n=3479] | Poorest | 1 | <0.001 | 1 | <0.001 |
|  | Intermediate | 0.69 (0.57 – 0.84) |  | 0.68 (0.55 – 0.83) |  |
|  | Wealthiest | 0.93 (0.76 – 1.14) |  | 0.93 (0.75 – 1.16) |  |
| **Assets index** [n=3479] | Poorest | 1 | 0.78 | – | – |
|  | Intermediate | 0.93 (0.77 – 1.13) |  | – |  |
|  | Wealthiest | 0.95 (0.78 – 1.15) |  | – |  |

**The first listed category of each variable has been taken as reference value.*

***Note****: CI – confidence interval; IPTp1+ – one or more intermittent preventive treatment doses; OR – odds ratio.*
